# Brain MRI before and at term equivalent age predicts motor and cognitive outcomes in very preterm infants

**DOI:** 10.1016/j.ynirp.2025.100262

**Published:** 2025-04-19

**Authors:** Alex M. Pagnozzi, Kerstin Pannek, Roslyn N. Boyd, Liza van Eijk, Joanne M. George, Samudragupta Bora, DanaKai Bradford, Michael Fahey, Michael Ditchfield, Atul Malhotra, Paul B. Colditz, Jurgen Fripp

**Affiliations:** a The Australian e-Health Research Centre, CSIRO, Brisbane, Australia; b The University of Queensland, School of Electrical Engineering and Computer Science, Brisbane, Australia; c Child Health Research Centre, Queensland Cerebral Palsy and Rehabilitation Research Centre, Faculty of Medicine, The University of Queensland, Brisbane, Queensland, Australia; d Department of Psychology, James Cook University, Townsville, Queensland, Australia; e Physiotherapy Department, Queensland Children's Hospital, Children's Health Queensland Hospital and Health Service, Brisbane, Australia; f Mothers, Babies and Women's Health Program, Mater Research Institute, Faculty of Medicine, The University of Queensland, Brisbane, Queensland, Australia; g Monash Health Paediatric Neurology Unit and Department of Paediatrics, School of Clinical Sciences, Monash University, Clayton, Victoria, Australia; h Monash Imaging, Monash Health, Melbourne, Victoria, Australia; i Department of Medicine, Monash University, Melbourne, Victoria, Australia; j Department of Paediatrics, Monash University, Melbourne, Victoria, Australia; k Monash Newborn, Monash Children's Hospital, Melbourne, Victoria, Australia; l Perinatal Research Centre, The University of Queensland Centre for Clinical Research, Faculty of Medicine, The University of Queensland, Brisbane, Queensland, Australia

**Keywords:** Biomarkers, Cerebral palsy, Magnetic resonance imaging, Neurodevelopmental impairment, Prediction, Preterm infants, Structural

## Abstract

Brain Magnetic Resonance Imaging (MRI) of high-risk infants in the neonatal period (from 26 weeks postmenstrual age to Term Equivalent Age (TEA)) is increasingly being used for the detection of brain injuries, and the early prognostication of adverse outcomes such as Cerebral Palsy (CP). While most imaging is performed around TEA in clinical practice for infants born preterm (<37 weeks of gestation), this would often require families to return to hospital for imaging. In this work, we extract structural biomarkers from MRI acquired both before and at TEA in a cohort of very preterm infants from the PPREMO and PREBO studies (n = 100), to determine if either time-point, or both combined, are predictive of both Bayley Scales of Infant and Toddler Development – Third Edition (Bayley-III) and the Neuro-sensory Motor Developmental Assessment (NSMDA) at 2 years. Using multivariable regression, moderately strong and statistically significant associations were found between brain structure on both early and TEA MRIs with 2-year outcomes (r = 0.39–0.55 for early MRI, r = 0.37–0.49 for Term MRI, r = 0.37–0.56 for early and TEA MRI combined). Importantly, brain biomarkers associated with early childhood outcomes from MRIs were identified, including white and grey matter volumes, deep grey matter and cerebellar volumes, and gyrification and surface area across the whole cortex. Early MRI showed the best prognostic accuracy along with combining timepoints, indicating the potential clinical benefit of Early MRI in predicting adverse outcomes.

## Highlights

• We used brain structure from very preterm infants to predict 2-year outcomes.
• MRI acquired before and at term equivalent age was compared in predictive accuracy.
• Brain tissue volume and gyrification predict motor and cognitive outcomes.
• Neuromorphometrics significantly predict motor and cognitive outcomes at 2-years.

## 1 Introduction

Preterm birth is the largest cause of death in newborn infants globally (Chang et al., 2013), and results in an increased risk of developmental delay (Pugliese et al., 2013). Increasingly, brain magnetic resonance imaging (MRI) acquired during the neonatal period, from 26 weeks postmenstrual age (PMA) up to term equivalent age (TEA), is improving the ability to detect evidence of brain injury, and plays an important role in identification of infants at risk of adverse motor and cognitive outcomes (George et al., 2018; Woodward et al., 2006), such as intelligence quotient (Anderson et al., 2017). Identifying early prognostic markers of later development and outcomes is crucial, given the enhanced neuroplasticity at this age, early interventions starting in the infant period may have the potential to significantly reduce motor and cognitive impairments (Spittle et al., 2015).

On structural MRI, preterm infants exhibit altered cortical morphometrics (Dubois et al., 2019; Kim et al., 2016; Shimony et al., 2016), growth and morphological trajectories (Bouyssi-Kobar et al., 2016; Lefèvre et al., 2016) compared to term born infants. Specifically at TEA, a number of studies have found MRI-derived measures to be associated with neurodevelopmental outcomes at 2 years and beyond (Anderson, 2011), including quantitative structural measures (such as volume, cortical shape), microstructural measures using diffusion MRI (Parikh, 2016) and qualitative measures such as white matter abnormality (van’t Hooft et al., 2015). In one study using the state-of-the-art developing Human Connectome Project (dHCP) structural pipeline (Makropoulos et al., 2018a) on TEA MRI data (Makropoulos et al., 2018b), anatomical volumes (for areas such as the thalamus) as well as increased cortical surface area and reduced cortical curvature were found to be associated with better 2-year Bayley-III motor and cognitive outcomes (Kline et al., 2020; Julia E. Kline et al., 2020). Furthermore, combining structural MRI measures with early assessments such as the General Movements Assessment (GMA) and Hammersmith Infant Neurological Examination (HINE) at 3–4 months corrected age (CA) have demonstrated stronger predictive accuracy for neurodevelopmental outcomes than TEA MRI alone (Setänen et al., 2014; Skiöld et al., 2013; Wang et al., 2021).

While much work on TEA imaging exists, it is unclear if performing imaging earlier, before TEA, provides similar or greater prognostic value. While early imaging (28–32 weeks PMA) has been performed in cohorts whose motor, cognitive and language trajectories were assessed up to 4.5 years of age, literature in this area is sparse. Using an MRI scoring system for white matter injury (WMI), the severe WMI classification from early MRI was associated with poor motor outcomes as measured by the MABC-2 at 4 years (Cayam-Rand et al., 2019). Using another semi-quantitative approach, WMI severity was strongly associated with neurodevelopmental impairments at 2 years (Spearman's rank correlation 0.88, p < 0.001) (Martinez-Biarge et al., 2019). In contrast, quantitative MRI morphology (tissue volumes) at the early time point (30 weeks PMA) and at TEA were found to be independently predictive of motor and cognitive impairment at around 2 years of age (Gui et al., 2019), with an area under the curve of 0.8 and 0.85 for low motor/cognitive outcomes respectively (Moeskops et al., 2017). Early imaging has the advantage being conducted prior to discharge, when the infant is still in the hospital, as part of early standard of care, often without the need for sedation or general anaesthesia. This timing could aid in the early identification and prediction of adverse motor or neurodevelopmental outcomes in infants born preterm and facilitate referral to early interventions while the infant is still in the hospital.

We previously demonstrated that brain volumetrics from early structural MRI predict motor and cognitive outcomes at 2 years of age (Pagnozzi et al., 2023). In the current study, we compared these findings with structural MRI measures at TEA in predicting motor and cognitive outcomes at 2 years CA, and then investigate the potential benefit of utilising both time points for this prediction. For this, we utilise the internationally unique prospective Prediction of PREterm Motor Outcomes (PPREMO) and Prediction of Preterm Brain Outcomes (PREBO) studies of infants born <31 weeks gestational age (GA) which together form one of the largest cohorts worldwide with 3T MRI acquired at a median postmenstrual age of 32 weeks (PMA; ‘early’) as well as at TEA (George et al., 2015a). These cohorts also underwent neurobehavioural and motor assessment concurrent with imaging at early and TEA time-points, and neurodevelopmental assessments at 2 years corrected age. By determining which time point has the best prediction of later outcomes (early, or TEA), we can determine what assessments at what times within the neonatal window are best for predicting motor and cognitive outcomes.

## 2 Methods

### 2.1 Participants

This study examined data from two longitudinal prospective cohort studies of very preterm infants: the Prediction of PREterm Motor Outcomes (PPREMO) study (George et al., 2015b) and the Prediction of childhood Brain Outcomes (PREBO) study (NHMRC1084032). Both the PPREMO and PREBO cohorts recruited preterm infants born at <31 weeks' GA with no congenital or chromosomal abnormality, whose parents/caregivers were English-speaking and lived within a 200 km radius of the recruiting hospital. The total number of preterm and term-born infants recruited was n = 146 for PPREMO and n = 187 for PREBO. Infants were recruited at the Royal Brisbane and Women's Hospital (RBWH), Monash Children's Hospital (MCH), and Mater Mothers Hospital (MMH) for PPREMO between February 2013 and February 2016 (RBWH only), and for PREBO between February 2016 and December 2019 (RBWH, MCH, MMH). Some PREBO participants received follow up including imaging at the Queensland Children's Hospital (QCH).

Ethics approval was obtained from the Royal Brisbane and Women's Hospital Human Research Ethics Committee (HREC/12/QRBW/245), the Royal Children's Hospital (HREC/15/QRCH/7), and The University of Queensland (2012001060, 2015000290) with reciprocal approval from the CSIRO Health and Medical HREC (2019_013_RR). Both cohort studies were registered with the Australian New Zealand Clinical Trials Registry (PPREMO: ACTRN12613000280707; PREBO: ACTRN12615000591550). Informed written parental consent was obtained for each infant.

### 2.2 Image acquisition

Infants were scanned at 29–35 weeks PMA and again at TEA during natural sleep using a 3T MRI with a dedicated neonatal head coil in an MR compatible incubator or paediatric head coil with no incubator (Table 1). PREBO infants were placed on an immobilisation pillow in the incubator to minimise movement. Noise from the MRI scanner was attenuated using mini muffs (Natus Medical Inc., San Carlos, CA). All infants were monitored with pulse oximetry and electrocardiographic monitoring. No sedation or anaesthesia was used. The PPREMO study used multi-echo T2-weighted turbo spin-echo (TSE) volumes acquired in the axial plane, while in the later PREBO study at RBWH, QCH and MCH, we acquired three orthogonal T2-weighted images (in axial, coronal, and sagittal plane) to improve image quality in relation to motion. Imaging parameters are detailed in Table 1 below.

**Table 1:** Scanner details and imaging parameters across the two studies and three sites.

Cohort	PPREMO	PREBO		
Site	RBWH	RBWH/QCH		MCH
MRI scanner	Siemens Tim Trio	Siemens Skyra		Philips Ingenia
Head coil	8-channel neonatal head coil, Lammers LMT incubator (first timepoint only)			32-channel head coil, Phillips
T2 sequence	T2 TSE	3x T2 HASTE		3x T2 SSh TSE
TR	10,580 ms	2280 ms		2280 ms
TE	27/122/189 ms	117 ms		117 ms
Flip angle	150	120		120
Field of view	144 × 180mm	SAG: 162.6 × 200mm COR: 137.6 × 200mm AX: 137.6 × 200mm		SAG: 160 × 200mm COR: 160 × 200mm AX: 160 × 200mm
Matrix	204 × 256mm	SAG: 208 × 256mm COR: 176 × 256mm AX: 176 × 256mm		SAG: 198 × 252mm COR: 197 × 252mm AX: 198 × 252mm
Voxel size	0.7 × 0.7 mm	0.8 × 0.8 mm		0.8 × 0.8 mm
Slice thickness	2 mm	1.8 mm		1.8 mm
Scan time	5:40 min	SAG: 3:27 min COR: 3:11 min AX: 2:53 min		SAG: 2:05 min COR: 1:08 min AX: 1:54 min

AX, Axial; COR, Coronal; LMT, Lammers Medical Technology; MCH, Monash Children's Hospital; RBWH, Royal Brisbane & Women's Hospital; SAG, Sagittal; TE, Echo Time; TR, Repetition Time; TSE, Turbo Spin Echo.

### 2.3 Image processing

We utilised the same MRI processing pipeline as in our previous work (Pagnozzi et al., 2023), including conversion of MRI, “thick slice” neonatal MRIs to a high-resolution image and segmentation using the state-of-the-art dHCP structural pipeline (Makropoulos et al., 2018b). Super-resolution reconstructed images and dHCP segmentations were visually inspected and rated as ‘failed’ (no reconstruction obtained), ‘poor’ (major artefacts/accuracy errors), ’good’ (minor artefacts/accuracy errors), or ‘excellent’ (no or negligible artefacts/accuracy errors). A quality rating of ‘good’ or ‘excellent’ was required for inclusion in further analysis. As the cohort included participants with intraventricular haemorrhage, in many cases of brain injury, specifically severely enlarged ventricles, resulted in an inaccurate segmentation. As a result, these participants where segmentation failed were excluded from the analysis. We extracted measures of cortical thickness (CT), sulcal depth (SD), surface area (SA) and gyrification index (GI) of each lobe of the cerebral cortex (frontal, parietal, temporal and occipital), as defined by the Gousias neonatal atlas (Gousias et al., 2013), and 8 tissue volumes (extracerebral CSF, cortical GM, WM, ventricles, cerebellum, DGM, brainstem, hippocampi/amygdala), resulting in a total of 24 variables. An example segmentation provided by the dHCP pipeline is illustrated in Fig. 1, for an early and TEA MRI.

**Fig. 1:**
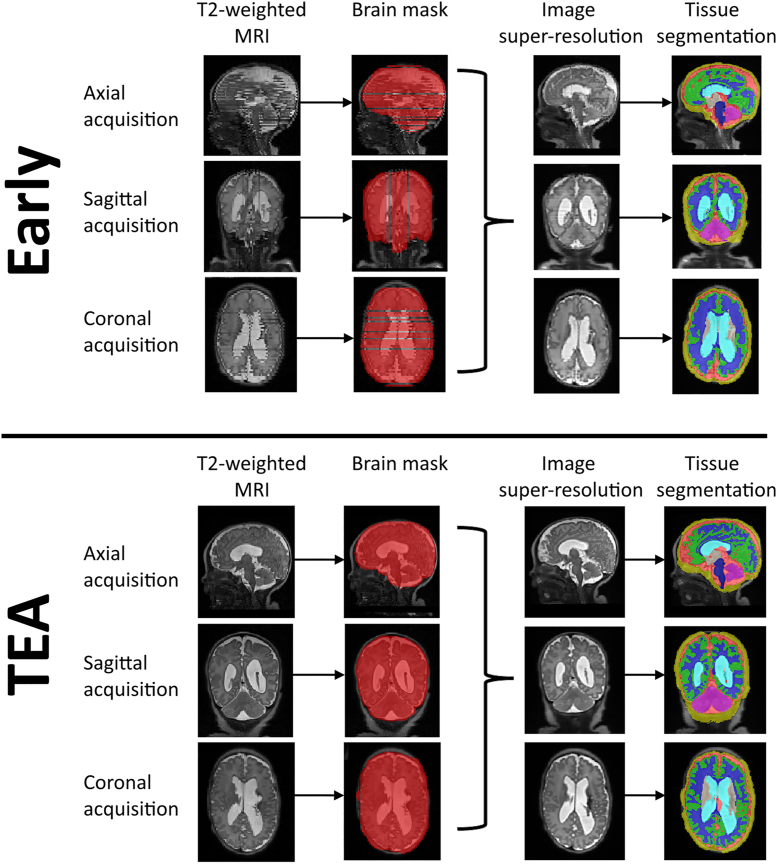
For both an ’early’ and ’TEA’ MRI of the same PREBO participant, the original T2 MRIs are shown (first column) in the sagittal, coronal and axial planes, respectively. These images underwent brain masking (shown in red in the second column), with slices containing artefact removed. These three acquisitions are combined into a single high-resolution MRI (third column), and this was then segmented using the dHCP pipeline with the tissue labels illustrated in the fourth column. dHCP, Developing Human Connectome Project; MRI, Magnetic Resonance Images; TEA, Term Equivalent Age.

### 2.4 Concurrent neonatal assessments

Infants were assessed at Early and TEA timepoints, and additionally at 3 months CA, with the General Movements Assessment (GMA). This assessment can be conducted from birth to 20 weeks CA (Olsen et al., 2018) and is a reliable tool for identifying infants at risk of adverse neurodevelopmental outcomes, including CP (Hadders-Algra et al., 2024). In the GMA, movement was classified as normal, poor repertoire, cramped synchronised, or chaotic in the writhing period, and as normal, abnormal fidgety movements, or absent fidgety movements in the fidgety period. In addition, a measure of socioeconomic status (SES) and parenting factors were collected through a questionnaire completed by infants’ primary care giver. This questionnaire provides a raw score from 0 to 12, with scores of 2 and above being considered high social risk (George et al., 2015a; Roberts et al., 2008) in line with other research in this population (see (George et al., 2015b), Supplementary Table 1).

### 2.5 Neurodevelopmental outcomes at 24 months corrected

Neurodevelopmental outcomes at 2 years CA were measured with the Bayley Scales of Infant and Toddler Development – Third Edition (Bayley-III) standardised assessment (Bayley, 2006), with the motor, cognitive and language composite scores being used (Armstrong and Agazzi, 2010; Crais, 2010). Motor outcome was further measured using the standardised Neuro-Sensory Motor Developmental Assessment (NSMDA) tool (Burns, 1992). These assessments were conducted by an experienced paediatric physiotherapist who was blinded to all earlier clinical and MRI findings. While the Bayley-III is a norm-referenced discriminate measure of developmental functioning of the infant (mean score 100,±15 standard deviation based on Australian infant data (Anderson et al., 2010)) with higher scores indicating better development, the NSMDA is a categorical score of motor performance, with scores that are normal for developmental age (6–8), and delayed skill attainment with scores ranging from: minimal motor dysfunction (9–11), mild (12–14), moderate (15–19), severe (20–25), and profound disability (>25). Both the Bayley-III and NSMDA have been shown to predict functional outcomes beyond 3 years of age (Boswell et al., 2017; Lowe et al., 2023).

### 2.6 Statistical analyses

Prior to associating MRI measures with 2-year outcomes and to minimise the risk of overfitting on our dataset, we investigated the collinearity of the extracted MRI measures using a correlation matrix. Highly collinear measures were then grouped if anatomically appropriate. Then the sparse, independent set of structural measures from MRIs taken at the early time point and TEA were associated separately and combined with 2-year outcomes using the Least Absolute Shrinkage and Selection Operator (LASSO) regression (Tibshirani, 1996). To further reduce the risk of model overfitting, the large number of structural measures, relative to the number of participants, was further reduced with model simplification (i.e. variable reduction) based on the L1 penalty. Key patient demographics and clinical variables were also included in all models as confounders, which include sex, GA at birth, PMA at MRI scans, socioeconomic status (raw score) and age at NSMDA assessment, as well as covariates for cohort (PPREMO/PREBO) to account for differences in the MRI acquisition and the demographics of the cohort itself, and MR image quality (which was visually classified as ‘good’, ‘fair’, ‘poor’ or ‘unusable’). Age at Bayley-III assessment was not included into any model as the measure inherently accounts for this. Variable reduction is performed implicitly in LASSO with a penalty term (alpha), with higher values enforcing sparser (fewer non-zero) model coefficients. Models were constructed using 30-week data and TEA data separately, as well as combining MRI measures from both timepoints. In addition, models with only confounders (i.e. no exposure MRI measures) were also constructed to determine the potential benefit of adding MRI information. All MRI measures were standardised prior to LASSO regression.

For each time point, complete data was split into the identical training (75 %) and test (25 %) sets. Models were generated on the training set, with the optimal sparsity term (alpha) determined using mean-square error and 5-fold cross validation. The alpha of the best performing model was then applied to the entire training set, yielding an optimal training model (which included only the retained features from LASSO as well as all confounders). This optimal training was applied to the test set, producing a Pearson's r correlation between actual versus predicted 2-year outcome as well as a mean absolute error (MAE). This was repeated for each clinical score, (Bayley motor, cognitive, language, and NSMDA), with each using the same train and test splits. Additionally, outcome measures on the test set were dichotomised as ‘normal’ or ‘poor outcome’, which for Bayley-III was defined as 1 standard deviation below the mean (scores <85) and for NSMDA as any functional grade indicating disability (scores >11). From this the Area Under the Curve (AUC) was quantified, which measured the overall classification agreement between the predicted model and the dichotomised outcome.

To investigate potential changes in brain growth observed among preterm-born infants ex-utero compared to term born controls (Bouyssi-Kobar et al., 2016), frequently MRI measures retained by LASSO were compared between preterm infants at the ‘early’ and ‘TEA’ timepoints, and term-born infants (at ‘Term’).

## 3 Results

### 3.1 Participants

**Fig. 2:**
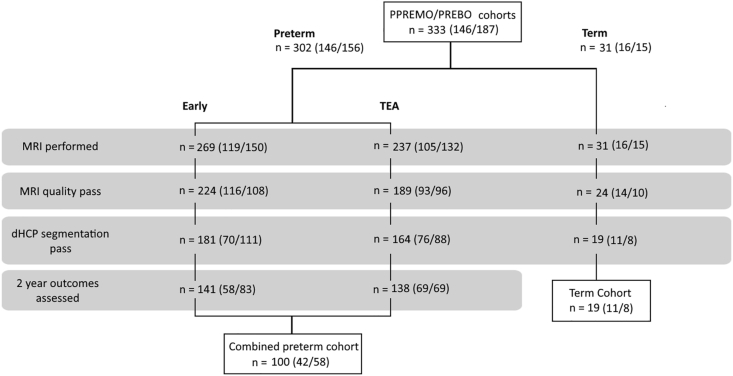
Diagram illustrating data available for statistical analysis, with reasons for exclusion. Number in brackets shows participants from each cohort (PPREMO/PREBO). dHCP, Developing Human Connectome Project; MRI, Magnetic Resonance Images; TEA, Term Equivalent Age.

Of the total number of participants in the PPREMO/PREBO cohorts (N = 333), the total number of participants available for analyses was n = 100 at both time points (see flowchart Fig. 2). This is accounting for poor MRI data quality (n = 62 early, 96 TEA) and/or segmentation errors (n = 47 early, 48 TEA) at either time-point, or missing assessment data at 2-years (n = 93), with the breakdown of data loss at each timepoint illustrated in Fig. 2. All of these participants formed part of the cohort of the our previous paper (n = 139) (Pagnozzi et al., 2023). Of these n = 100 infants, n = 42 were part of the PPREMO study and 58 were part of the PREBO (RBWH) study. The overall demographics, clinical assessments and measures of care of the preterm infants are detailed in Table 2 below. While there was only one case of confirmed sepsis in this cohort (1 %), thirteen infants underwent surgery (13 %), and there was a high incidence of chorioamnionitis during pregnancy (16 %) as well as intraventricular haemorrhage (30 %) as observed on cranial ultrasound. The demographics of the included participants in the analysis and the excluded participants are in Supplementary Table 1, with the excluded cohort having a significantly greater GA (1 week median difference, p = 0.04) and an increased incidence of hydrocephalus (n = 9, p = 0.03), sepsis (n = 11, p < 0.01) and endotracheal tube ventilation (p = 0.04).

**Table 2:** Baseline characteristics of the very preterm cohort with MRI at both early and TEA timepoints with 2-year follow-up assessments. Characteristics of the term-born infants (scanned only at TEA) used to compare structural biomarkers with the preterm cohort is also given below.

	Preterm Cohort (n = 100)		Term-born (n = 19)
MRI PMA early, median weeks ^+ days^ (range)	32^+4^ (29^+2^ - 36^+2^)		NA
MRI PMA TEA, median weeks ^+ days^ (range)	41^+6^ (38^+3^ - 45^+2^)		41^+6^ (39^+3^ - 44^+5^)
Gestational age, median weeks ^+ days^ (range)	28^+2^ (24^+3^ - 30^+6^)		39^+6^ (38^+4^ - 41^+0^)
Male, n (%)	44 (44 %)		7 (37 %)
Socioeconomic status, mean (% at risk, >2)	1.71 (25 %)		0.21 (0 %)
GMs classification at 30w and TEA			
Normal	42	41	–
Poor Repertoire - writhing period	53	46	–
Cramped Synchronised - writhing period	3	6	–
Chaotic - writhing period	0	0	–
Missing	2	7	–
Bayley motor composite at 2 years CA (SD)	96 (23)		NA
Bayley cognitive composite at 2 years CA (SD)	98 (16)		NA
Bayley language composite at 2 years CA (SD)	92 (22)		NA
NSMDA functional grade at 2 years CA			
Normal for developmental age (6–8)	67		NA
Minimal motor dysfunction (9–11)	22		NA
Mild motor dysfunction (12–14)	10		NA
Moderate motor dysfunction (15–19)	0		NA
Severe motor dysfunction (20–25)	1		NA
Profound disability (>25)	0		NA
Observed brain injury			
Hydrocephalus, n (%)	1 (1 %)		NA
Periventricular leukomalacia, n (%)	1 (1 %)		NA
Intraventricular haemorrhage, n (%)	30 (30 %)		NA
Grade I, n	19		NA
Grade II, n	7		NA
Grade III, n	1		NA
Grade IV, n	3		NA
Clinical characteristics			
Clinical Chorioamnionitis, n (%)	16 (16 %)		NA
Confirmed sepsis, n (%)	1 (1 %)		NA
Days until discharge, days, median (SD)	74 (34)		NA
Clinical measures of care			
Surgery, n (%)	13 (13 %)		NA
Antenatal corticosteroids, n (%)	68 (68 %)		NA
Days of ETT ventilation, days, median (SD)	3 (10.1)		NA
Days of CPAP, days, median (SD)	16 (13.1)		NA
Hours of oxygen therapy, hours, median (SD)	54 (390.2)		NA
Days of TPN, days, median (SD)	11 (5.2)		NA
Hours of phototherapy, hours, median (SD)	51 (53.7)		NA

CA, corrected age; CPAP, Continuous Positive Airway Pressure; ETT, Endotracheal Tube; GMs, General Movements assessment; NSMDA, Neuro-Sensory Motor Developmental Assessment; PMA, Postmenstrual age; SD, Standard deviation; TEA, Term Equivalent Age; TPN, Total Parenteral Nutrition.

### 3.2 Correlation of structural measures

**Fig. 3:**
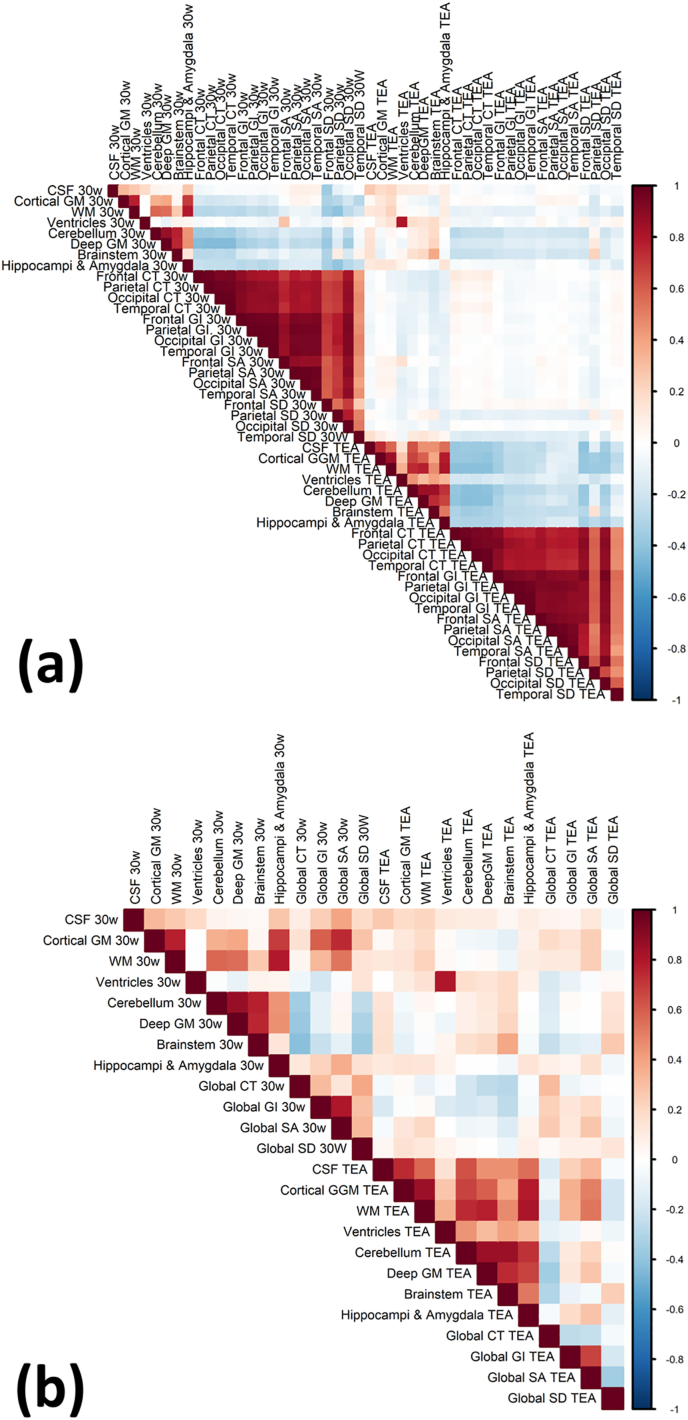
Correlation matrix of (a) all structural measures, and (b) manually reduced feature set using global measures of cortical shape. CSF, Cerebrospinal Fluid; CT, Cortical Thickness; GI, Gyrification Index; GM, Grey Matter; SA, Surface Area; SD, Sulcal Depth; WM, White Matter; TEA, Term Equivalent Age.

All 24 structural measures (8 vol, 16 cortical shape) at each of the two timepoints were put into a correlation matrix (Fig. 3a). Independent variables with a Pearson's correlation above 0.9 are highly correlated and can be reduced or combined. We observed that all cortical measures were highly correlated with each other, independently for both the ‘early’ (labelled 30w) and ‘TEA’ timepoints. To reduce model complexity and for the feature set to remain anatomically consistent, all cortical measures were grouped from a frontal, temporal, occipital and parietal lobule average score to a global average. This reduced the number of structural measures from 48 to 24 (12 at each time point), and this reduced set of structural features showed markedly reduced collinearity (Fig. 3b). Furthermore, to test the importance of cortical shape, volume-only models were also constructed and validated on the same training and testing sets.

### 3.3 Structural brain measures impacting 2-year Bayley-III composite scores and NSMDA

For each of the 12 models (4 outcome assessments, each using 2 MRI time points individually and combined), the features were found to be associated with changes in 2-year outcomes and hence were retained from LASSO regression. The coefficients for the ‘early’ and ‘TEA’ models are provided in Table 3. With the exception of the NSMDA models, most structural measures were retained from LASSO. In all cases, confounders were specifically retained by LASSO. Table 3 outlines the coefficient of each retained structural measure, and at which time point, and the model fit provided by the pseudo R2 for the complete model and for models without MRI features (i.e. only clinical variables). In all models the pseudo R2 of models with MRI features was increased compared to the clinical variables only models, indicating the MRI measures independently explained variance in the outcomes. Structural biomarkers that are associated with better 2-year outcomes will show a positive association for the Bayley-III composite sores, and negative associations for NSMDA. This is because while higher Bayley-III scores indicate better outcomes, higher NSMDA scores indicate greater dysfunction.

**Table 3:** Morphometric biomarkers and their association with 2-year Bayley-III composite scores and NSMDA functional grade. The MRI (exposure) variables are shown above the included confounders. Model fit (pseudo R^2^) with and without the MRI exposure variables is presented at the bottom of the table. Exposure variables dropped by LASSO are designated ‘-‘.

	Bayley motor composite score		Bayley cognitive composite score		Bayley language composite score		NSMDA functional grade	
Feature	Early coefficient	Term coefficient	Early coefficient	Term coefficient	Early coefficient	Term coefficient	Early coefficient	Term coefficient
Extracerebral CSF	−10.4	−5.41	−1.85	−0.91	−6.73	−4.72	0.29	3.20
Cortical grey matter	16.6	13.7	–	3.16	10.7	5.43	−0.16	–
White matter	–	10.1	2.82	4.05	–	4.47	−0.92	–
Ventricles	−1.48	−3.45	−1.27	−5.19	−1.60	–	–	0.09
Cerebellum	–	5.38	1.41	−1.05	10.9	3.47	–	–
Deep grey matter	8.51	0.53	8.35	–	5.68	5.89	−0.28	−0.26
Brainstem	11.8	5.86	–	4.11	−9.23	7.86	–	−0.76
Hippocampus/amygdala	−7.07	1.04	5.90	–	−10.3	−0.51	–	−0.12
Global CT	−0.28	−4.86	−2.07	−3.14	−0.85	−2.51	–	0.25
Global GI	−0.01	9.59	0.13	−2.17	1.61	2.96	–	−1.03
Global SA	–	−0.07	1.05	0.007	9.70	−6.42	–	–
Global SD	0.22	10.3	1.24	−0.58	1.83	−0.99	−0.29	−0.83

Sex (REF: Male)	0.85	1.99	2.88	7.69	9.96	2.35	0.44	0.54
GA at birth	0.64	0.35	0.15	0.12	0.49	0.35	−0.007	0.009
PMA at MRI	−6.42	−0.47	−2.47	−0.13	−7.12	0.35	0.59	−0.02
SES	−2.11	−6.23	−3.59	−2.39	−2.54	−4.12	0.30	0.28
GMs classification	0.05	0.05	−0.02	−0.005	0.04	0.08	0.001	0.04
Cohort (REF: PPREMO)	11.3	13.4	0.83	0.66	6.11	5.16	−0.008	−0.56
Image quality	0.05	0.01	0.08	0.01	0.03	0.02	0.003	0.03

**Pseudo R2**	0.813	0.799	0.811	0.799	0.807	0.709	0.919	0.722

**Pseudo R2 (no MRI)**	0.215	0.203	0.230	0.217	0.260	0.176	0.142	0.258

CSF; CT, Cortical Thickness; GA, Gestation Age; GI, Gyrification Index; MRI; Magnetic Resonance Imaging; PMA, Postmenstrual age; SA, Surface Area; SD, Sulcal Depth; SES, Socio-economic status.

Cortical grey matter was positively associated with better outcomes in most models (5 of 6), and conversely larger ventricles were consistently associated with poorer outcomes across 5 of the 6 models. White matter volume was generally positively associated with better outcomes (in 4 of 6 models), and similarly cerebellum volume was positively associated with better outcomes in 4 of 6 models. The remaining structural measures were more mixed in terms of association with outcome; however we note that gyrification index was positively associated with higher Bayley motor, cognitive and language composite scores, whilst sulcal depth was positively associated with improved Bayley scores and NSMDA grade. Extracerebral CSF (in all 6 models) and ventricular CSF (in 5 of 6 models) was negatively associated with Bayley outcomes, which was also the only finding consistent with the univariate analysis that did not adjust for confounders (Supplementary Table 2, Supplementary Fig. 1).

Higher social risk was negatively associated with all Bayley composite scores (average reduction −3.50 per SES risk level, accounting for MRI measures). Higher GA at birth was associated with better outcomes (score increase 0.35 per week). PMA at early MRI was negatively associated with Bayley outcomes (score decrease 5.34 per week), however the PMA at Term MRI was not strongly associated with 2-year outcomes (score decrease of 0.08 per week). As shown previously (Pagnozzi et al., 2023), females performed better than males across all models (average increase 4.29), and PREBO participants had higher Bayley outcomes at 2 compared to PPREMO participants (average increase 6.24). There was little association between Bayley outcomes and the included confounders; GMAs classification at the early (0.02) and TEA timepoint (0.04), and image quality (0.03).

### 3.4 Prediction model validation and classification performance

The trained LASSO models were used to predict assessments scores for the unseen test data (25 %). Pearson's r correlations between actual and predicted assessment scores are provided in Table 4. Moderate to strong correlations were observed for all assessments, with all but two statistically significant in the test set when accounting for multiple comparisons (Bonferroni correction *p* < 0.05/12 tests = 0.004). All models using structural measures from the early MRI, and both early and TEA MRI combined, were significant in the test set, with the combined model showing the greatest predictive accuracy in all cases except the Bayley-III language score. When looking at measures of volume alone, excluding measures of cortical shape which are more computationally difficult to obtain, predictive performance was overall slightly reduced on the test set (Supplementary Table 3).

**Table 4:** Correlations between the best performing models and the test set assessment scores unseen by the model.

	Early MRI		Term MRI		Combined	
	Pearson's r	MAE	Pearson's r	MAE	Pearson's r	MAE
Bayley motor composite	0.480∗ (p = 0.0004)	14.14	0.496∗ (p = 0.0002)	18.67	**0.561∗∗ (p < 0.0001)**	**16.96**
Bayley cognitive composite	**0.551∗∗ (p < 0.0001)**	**10.87**	0.456∗ (p = 0.0008)	12.11	0.492∗ (p = 0.0004)	14.99
Bayley language composite	0.399 (p = 0.004)	16.41	0.373 (p = 0.007)	16.92	0.373 (p = 0.004)	15.11
NSMDA functional grade	0.511∗ (p = 0.0001)	1.84	0.435∗ (p = 0.0016)	2.22	**0.534∗∗ (p < 0.0001)**	**2.04**

Bold indicates the timepoints with best statistically significant prediction accuracy.

∗*p* < 0.004, ∗∗*p* < 0.0001. MRI, Magnetic Resonance Images; NSMDA: Neuro-sensory Motor Developmental Assessment.

**Fig. 4:**
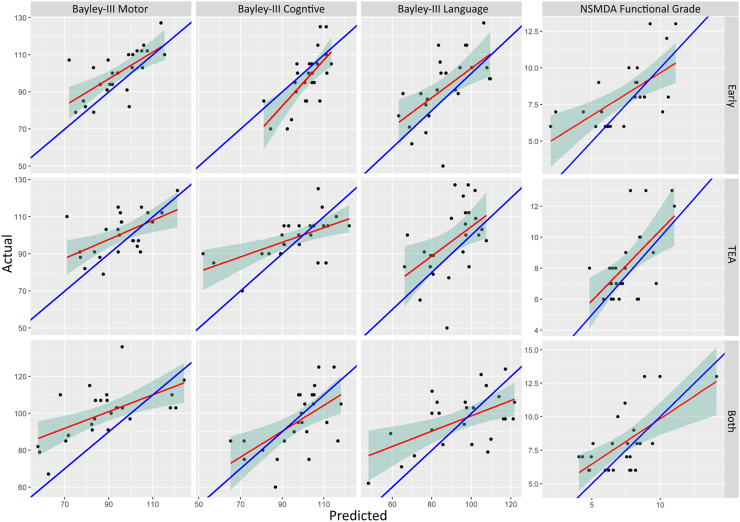
Test set correlations between the predicted outcome from the best performing LASSO model and the actual Bayley III assessment scores. Columns illustrate; Bayley-III motor composite score, first column; cognitive composite score, second column; language composite score, third column; and NSMDA (fourth column) using measures from the early MRI (first row), TEA MRI (second row) and both time points combined (third row). The regression line is shown in red, and the y = x line is shown in blue. LASSO, Least Absolute Shrinkage and Selection Operator; MRI, Magnetic Resonance Images; NSMDA, Neuro-sensory Motor Developmental Assessment; TEA, Term Equivalent Age.

In addition, the sensitivity, specificity and AUC metrics for classifying mild developmental delay from Bayley composite scores (<1 SD below mean, scores <85) (Celik et al., 2020) and disability from the NSMDA functional grade (scores >11) are shown in Table 5. The scatter plots of the model predicted score versus the actual score are illustrated in Fig. 4.

**Table 5:** Area Under the Curve (AUC), sensitivity and specificity metrics indicating agreement between the best performing models and the test set assessment scores unseen by the model.

	Early MRI			Term MRI			Combined		
	AUC	Sens (%)	Spec (%)	AUC	Sens (%)	Spec (%)	AUC	Sens (%)	Spec (%)
Bayley motor composite	0.84	90	80	0.82	78	100	0.88	85	100
Bayley cognitive composite	0.82	72	86	0.58	95	50	0.84	100	78
Bayley language composite	0.87	83	86	0.78	78	85	0.75	95	50
NSMDA functional grade	0.95	100	91	0.91	100	71	0.98	100	95

Bolded values are the time-point with the highest AUC metric for each outcome.

AUC, Area Under the Curve; MRI, Magnetic Resonance Images; Sens, Sensitivity; Spec, Specificity; NSMDA: Neuro-sensory Motor Developmental Assessment.

### 3.5 Comparing ex-utero brain development of preterm-born infants to term-born infants

**Fig. 5:**
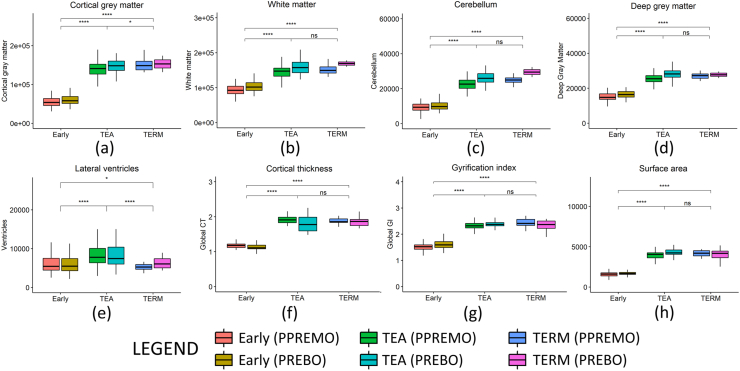
Box and whisker plots of several extracted structural measures comparing the preterm cohort acquired early and at TEA, and the term-born cohort acquired at TEA (TERM). PPREMO and PREBO cohorts are plotted separately, however statistics are only compared between ‘early’, ‘TEA’ and ‘TERM’ groups. CT, Cortical thickness; GI, Gyrification index; SA, Surface area; TEA, Term Equivalent Age.

For the frequently retained structural measures obtained from LASSO, the measures were compared between the preterm infants at the ‘early’ and ‘TEA’ timepoints (n = 100), as well as the term-born infants (n = 19) (Fig. 5). In most cases, the significant difference we see at the ‘early’ timepoint largely disappears at TEA, with the preterm infants showing no significant difference from the term-born group. This is not true for cortical grey matter however, which shows a slight but significant reduction in volume at term compared to term-born infants (7.5 %). While this difference is not reflected in cortical thickness, we note that the range of the cortical thickness measured was biologically congruent (1.3 mm at 28 weeks of PMA to 1.8 mm at term equivalent) (Liu et al., 2021). Also due to the presence of ventriculomegaly in the preterm cohort, lateral ventricle volumes are significantly larger at TEA compared to the term group. We also observed differences in volume between the PPREMO and PREBO studies, with PREBO reporting larger cortical GM (2.5 and 4.3 % at Early and TEA time points respectively), WM (7.8 and 11.8 %), cerebellar (2.0 and 13.4 %) and deep GM (8.5 and 10.5 %), which likely reflect the differences in image acquisition (Table 1).

## 4 Discussion

Measures from structural MRI taken at <32 weeks PMA (‘early’) and at TEA are associated with motor, cognitive and language ability at two-years CA in a very preterm cohort. Twelve LASSO models were generated, one for each of the four 2-year outcomes, and one for each timepoint (early, TEA, and both combined). By combining brain morphometrics with key clinical confounders using LASSO, ten of the twelve investigated models demonstrated moderate associations with 2-year functional outcomes in an independent test set (25 % of available data) unseen by the model. This study demonstrates that automated structural biomarkers from TEA MRI is predictive of 2-year outcomes (AUC 58–82 %), albeit showed lower prognostic accuracy than biomarkers from the early MRI (AUC 82–87 %), which is similar to our previous findings (Pagnozzi et al., 2023). Furthermore, there is some independence between the time-points as both timepoints combined led to the highest predictive performance for motor outcomes (Table 4). LASSO regression revealed volumes of the CSF, cortical GM, WM and cerebellum were consistently associated with outcomes at 2 years CA (Table 3), which expands on the list of structural biomarkers found in previous work on this cohort that utilised MRI scoring methods at both early and TEA time-points (George et al., 2021).

This indicates the potential value of quantitative analysis in clinical practice, specifically at the early time-point, for the prediction of long-term motor and cognitive outcomes (Hinojosa-Rodríguez et al., 2017). In terms of implications for clinical practice, this research suggests that acquiring early MRI in the first weeks of life, in addition to the TEA MRI (>38 weeks) typically performed in clinical practice, may more fully characterise brain structure and the trajectory of brain development (AUC = 84 %) (Inder et al., 2021).

While most structural measures were retained at both time points, we observed a consistent volumetric effect of increased GM and WM volume, and decreased extracerebral CSF and ventricle volume, to be associated with improved 2-year outcomes. This is consistent with findings in the literature comparing preterm and term-born infants at TEA (Kelly et al., 2020; Thompson et al., 2007), and reflects that underlying cerebral GM/WM dysmaturation leads to poorer outcomes (Inder et al., 2005). Similarly we found that greater deep grey matter and cerebellar volume, specifically at the TEA time-point, is positively associated with improved outcomes, which has previously been shown to predict neurodevelopmental outcomes at 4 years of age (Young et al., 2015). In terms of cortical morphology, gyrification index was found to be associated with better Bayley-III scores which has been shown previously (Julia E Kline et al., 2020), while cortical thickness at TEA was negatively associated with better Bayley-III scores. This latter result has been shown in previous morphological/microstructural studies, finding greater cortical thickness in frontal, insular and anterior parietal cortices in preterm infants compared to term-born infants at TEA (Dimitrova et al., 2021). At both time-points, surface area was positively associated with neurodevelopmental outcomes at 2-years of age which has been observed previously (Julia E Kline et al., 2020; Julia E. Kline et al., 2020). Despite grouping of structural measures, both gyrification index and sulcal depth as well as surface area reflect some of the same anatomical features, suggesting there may still be some collinearity in the models and further variable reduction may improve model performance. Interestingly, while LASSO aims to reduce the number of structural biomarkers, in all models most structural markers were retained, indicating a more global impact of brain structure on developmental outcome (Thompson et al., 2005). This could, in part be due to our efforts to retain independent structural features through grouping cortical features.

As we found in our previous work (Pagnozzi et al., 2023), higher social risk was negatively associated with poorer motor, cognitive and language outcomes, and was one of the biggest drivers we found of 2-year outcomes. Social risk is known to be a strong factor impacting childhood development (Dalmaijer et al., 2023). Further, we observed GMAs classification to be weakly, positively associated with Bayley composite scores, in contrast to our previous work (average reduction −1.01 per early GMAs score). The authors note that 3-month GMAs have been found to be the most predictive of later outcomes (John et al., 2022). Interestingly while PMA at Term MRI was not strongly associated with 2-year outcomes (score decrease of 0.08 per week), PMA at early MRI was negatively associated with Bayley outcomes (score decrease 5.34 per week) i.e. a later “early” MRI was associated with lower Bayley outcomes. The authors speculate that this could be due to babies with more complex clinical needs having their early MRI delayed until they were clinically stable enough to undergo MRI. These babies are also more likely to have poorer outcomes; potentially explaining the observed association between PMA at early MRI and 2-year outcomes. Alternatively, this may be due to collinearity between brain size and development at this time point and PMA at this time point, the latter of which was manually retained in all LASSO models. Brain structure of the very preterm infants at TEA did not significantly differ from the term-born controls (Fig. 5), with the exception of cortical grey matter and the lateral ventricles which are a reflection of underlying brain injury (Agut et al., 2020). This does not support the observed volumetric differences between preterm-born infants ex-utero compared to term born controls (Bouyssi-Kobar et al., 2016), however we note the limited statistical power in being able to detect potential differences due to the small term-born cohort (n = 19).

Further, using these structural biomarkers we were able to classify mild/moderate/severe motor and cognitive developmental delay with an AUC of 0.84 with early MRI, 0.82 with TEA MRI, and 0.88 combined (Table 5). This result is slightly improved on our previous deep-learning approach to detect mild motor delay on a smaller sample from the same cohort (AUC 0.72, n = 77) (Saha et al., 2020), and similar to a structural MRI study utilising TEA MRI (AUC 0.78 for low cognitive outcome, and an AUC 0.80 for low motor outcome) (Moeskops et al., 2017). These findings are consistent with similar research utilising early MRI for the identification of white matter injury (AUC 0.75 for mild motor delay, and AUC 0.8 for mild cognitive delay), which was observed at a later timepoint (4.5 years) suggesting this developmental delay continues through early childhood (Cayam-Rand et al., 2019). We show a similar classification accuracy of mild language delay (AUC 0.80) to mild motor and cognitive delay, which is lower than previous research (AUC 0.85) (He et al., 2021) which instead utilised a multi-modal MRI (structural, diffusion and functional MRI) to leverage both the structural and functional connectome for prediction. When looking at diagnostic accuracy, obtained sensitivity (72–100 %) and specificity (50–100 %) are on par with previous findings using TEA MRI to predict 18–24 month CA outcomes (sensitivity 33–100 %, specificity 30–97 %) (Banihani et al., 2021). Overall, we found that both early and TEA MRI was able to classify normal vs. mild/moderate/severe motor, cognitive and language delay. This was observed looking at both timepoints independently, as well as combined which led to slightly improved classification performance (Table 5).

There are several limitations to the present study, the first being the variability in the structural sequences used due to recruitment spanning two separate projects over 7 years. There was variation introduced by sequence specific preprocessing for the T2-TSE and T2 HASTE sequences to conform with processing of the dHCP structural pipeline. As a result, both image quality and sequence (cohort) were included as confounders in the LASSO analysis to account for this difference. Further, we note that both sequences have a smaller slice thickness (<2 mm) than a standard clinical T2 HASTE sequence. As such the pipeline remains to be validated on clinical data, and additionally to leverage the T1w sequence to better define tissue boundaries and to explore myelination through the T1w/T2w ratio (Soun et al., 2017). However, we found the T1w sequences to be of poorer image quality in our cohort, largely due to motion artefact caused by the long duration of the T1w scan (∼5 min), and hence these were not included in the analysis. Additionally, there are several methodological improvements that could be performed to improve robustness to more severe image artefact or brain injury. More advanced image super-resolution approaches now exist leveraging deep learning to interpolate accurate anatomical detail for images with slice thickness as high as 6.5 mm (Delannoy et al., 2020; Li et al., 2021). Such approaches can utilise variable slice selection to avoid sampling from motion affected slices (Sui et al., 2021), which unlike the current approach could accommodate multiple adjacent slices impacted by motion. Furthermore, deep learning segmentation approaches, such as iBEAT V2.0 (Wang et al., 2023) have shown remarkable performance compared to other neonatal pipelines. Such an approach may prove effective for MRIs with severe brain injury, which typically failed with the dHCP structural pipeline due to its disparity to the age-matched healthy atlas, and thus were largely excluded from the current analysis. Finally, there are logistical challenges in performing early MRI in very preterm cohorts in most clinical settings, requiring more careful monitoring of the infant and dedicated hardware (neonatal head coil, incubator).

Future work will incorporate measures of microstructure from diffusion MRI in addition to structural MRI as part of the prediction models (Dubois et al., 2021), to enhance the prediction of long-term outcomes. Associations between diffusion tensor imaging (DTI), yielding measures of fractional anisotropy and mean diffusivity, and neurodevelopmental outcomes at 3 years have been found (Kidowaki et al., 2017). More advanced diffusion models such as fixel-based analysis (Raffelt et al., 2017) provided advantages over simpler models such as DTI in their ability to resolve crossing fibres, and demonstrated stronger associations with motor and cognitive outcomes compared to DTI on the same cohort (Pannek et al., 2020). The authors also note that a limitation of the present study was the included cohort had a relatively low incidence of adverse outcomes (n = 15 infants had a Bayley motor composite score <1 SD below the mean, only n = 1 had severe motor dysfunction according to the NSMDA). As such, in future work the prediction models presented here will be validated on an external cohort to determine their generalisability to the wider preterm population. We also intend to apply these prediction models beyond 2 years CA, to reveal the utility of each imaging time-point in predicting later neurodevelopment (beyond 3 years CA), when intellectual, learning and behavioural delay can be fully determined (Arcangeli et al., 2012). This cohort is currently being followed up at 6 years of age with both advanced neuroimaging and concurrent clinical assessment of motor, neurological and neurobehavioural function as part of the PREBO-6 study (George et al., 2020). At this later time-point, a diagnosis of CP, ASD and school readiness outcomes can be reliably obtained, and can elucidate the long-term predictive value of early and TEA neonatal MRI.

## 5 Conclusions

We have leveraged state-of-the-art structural image analysis on our large, multi-centre, regional cohort of 100 very preterm infants, who underwent 3T MRI imaging at early and Term time points. Using LASSO regression and a model validation strategy to minimise overfitting, we found strong and significant association with motor, cognitive and language function at 2 years CA. We revealed consistent volume differences in the white and grey matter, deep grey matter and cerebellum, as well as surface area and gyrification of the cortex, associated with improved 2-year outcomes. In addition to key patient demographics and clinical variables, including socio-economic status, GMs assessment classification, sex and GA at birth, these biomarkers revealed both time-points are independently predictive of outcomes, suggesting either could be used in clinical practice. Early MRI showed the best prognostic accuracy compared to TEA MRI, indicating that imaging preterm infants before TEA not only help ensure at-risk infants are scanned prior to discharge from hospital, but that brain structure at this time may better identify at-risk infants. These early biomarkers are key to provide targeted interventions to at-risk preterm infants much earlier, when they are most effective.

## CRediT authorship contribution statement

**Alex M. Pagnozzi:** Writing – review & editing, Writing – original draft, Visualization, Validation, Software, Resources, Methodology, Investigation, Data curation. **Kerstin Pannek:** Writing – review & editing, Supervision, Methodology, Data curation, Conceptualization. **Roslyn N. Boyd:** Writing – review & editing, Project administration. **Liza van Eijk:** Software, Methodology, Investigation, Data curation. **Joanne George:** Writing – review & editing, Project administration, Funding acquisition, Data curation, Conceptualization. **Samudragupta Bora:** Writing – review & editing, Supervision, Project administration, Funding acquisition. **DanaKai Bradford:** Writing – review & editing, Supervision. **Michael Fahey:** Writing – review & editing, Data curation. **Michael Ditchfield:** Writing – review & editing, Data curation. **Atul Malhotra:** Writing – review & editing, Data curation. **Paul B. Colditz:** Writing – review & editing, Project administration, Funding acquisition, Data curation, Conceptualization. **Jurgen Fripp:** Writing – review & editing, Supervision, Project administration.

## Funding

The following support has been provided by the Australian 10.13039/501100000925National Health and Medical Research Council (10.13039/501100000925NHMRC): Project grant (PREBO 10.13039/501100000925NHMRC 1084032) and Investigator Fellowship (RB: 10.13039/501100000925NHMRC 1195602) and an 10.13039/501100000925NHMRC Clinical CRE (10.13039/501100000925NHMRC 1116442 CRE 10.13039/501100022212Australasian CP Clinical Trials Network). PPREMO was funded through grants from the 10.13039/100012256Cerebral Palsy Alliance Research Foundation (IRG1413), the Financial Markets Foundation for Children (2014–074), the 10.13039/501100003550Queensland Government (Smart State; Health Practitioner Stimulus Grant) and the Royal Brisbane and Women's Hospital.

## Declaration of competing interest

The authors declare that they have no conflict of interest.

## Figures and Tables

**figs1:**
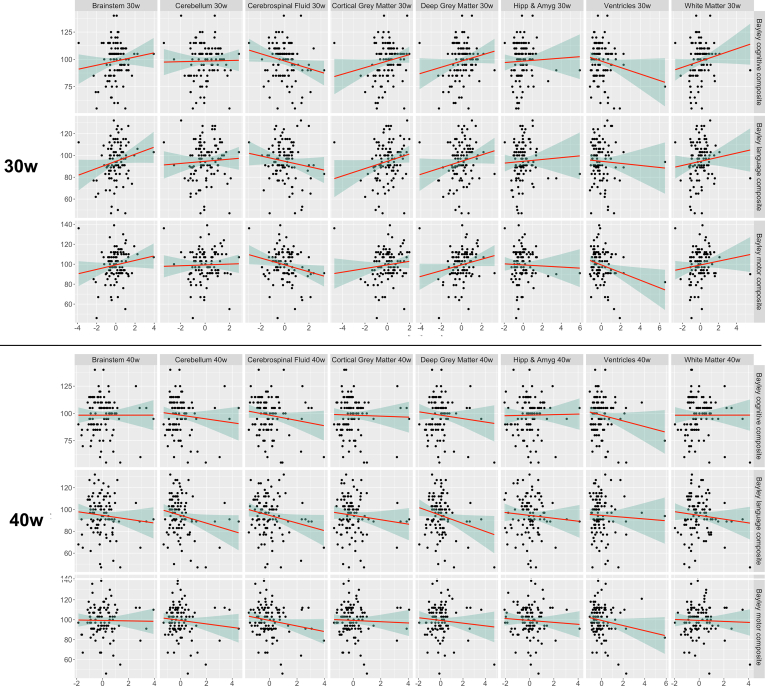


## Data Availability

The authors do not have permission to share data.
