# Supplementary material for: Brain MRI before and at term equivalent age predicts motor and cognitive outcomes in very preterm infants
Source: Neuroimage Rep. 2025 Apr 19;5(2):100262. doi: 10.1016/j.ynirp.2025.100262 (PMC12172852; doi:10.1016/j.ynirp.2025.100262)
Supplement: Multimedia component 1 [file mmc1.docx]

## Suggested reviewers

1. Serena Counsell

[serena.counsell@kcl.ac.uk](mailto:serena.counsell@kcl.ac.uk)

Professor of Perinatal Imaging & Health in the School of Biomedical Engineering & Imaging Sciences.

1. Rod Hunt

[Rod.Hunt@monash.edu](https://research.monash.edu/en/persons/rod-hunt)

Paediatrics Monash Health

1. Andreas Schuh

[andreas.schuh@imperial.ac.uk](mailto:andreas.schuh@imperial.ac.uk)

1. Peter J Anderson

[peter.j.anderson@monash.edu](mailto:peter.j.anderson@monash.edu)

Monash Institute of Cognitive and Clinical Neurosciences, Monash University, Melbourne, Victoria, Australia; Clinical Sciences, Murdoch Childrens Research Institute, Melbourne, Victoria, Australia.
