# Supplementary material for: Brain MRI before and at term equivalent age predicts motor and cognitive outcomes in very preterm infants
Source: Neuroimage Rep. 2025 Apr 19;5(2):100262. doi: 10.1016/j.ynirp.2025.100262 (PMC12172852; doi:10.1016/j.ynirp.2025.100262)
Supplement: Multimedia component 2 [file mmc2.docx]

# Supplementary Material

Supplementary Table 1. Demographics and 2-year outcomes of the very preterm cohort included in the analysis, and those that were not included due to MRI or segmentation quality issues (at either MRI timepoint), or not being followed up at 2 years.

|  | Included preterm cohort  (n=100) | Excluded preterm cohort  (n=202) | P-value |
| --- | --- | --- | --- |
| MRI PMA early, weeks^+days^, median (range) | 32^+4^ (7^+0^) | 32^+4^ (5^+6^) | 0.78 |
| MRI PMA TEA, weeks^+days^, median (range) | 41^+6^ (7^+2^) | 41^+6^ (8^+2^) | 0.13 |
| Gestational age, weeks^+days^, median (range) | 28^+2^ (6^+4^) | 28^+2^ (7^+5^) | 0.046* |
| Male, n (%) | 44 (44%) | 120 (59%) | 0.18 |
| Socioeconomic status, mean (% at risk) | 1.71 (25) | 1.78 (22) | 0.63 |
| Bayley motor composite at 2 years CA (SD) | 96 (23) | 92 (15) | 0.18 |
| Bayley cognitive composite at 2 years CA (SD) | 98 (16) | 92 (14) | 0.21 |
| Bayley language composite at 2 years CA (SD) | 92 (22) | 85 (25) | 0.19 |
| Observed brain injury |  |  |  |
| Hydrocephalus, n (%) | 1 (1%) | 9 (4.5%) | 0.03* |
| Periventricular leukomalacia, n (%) | 1 (1%) | 7 (3.5%) | 0.09 |
| Intraventricular hemorrhage, n (%) | 30 (30%) | 43 (21%) | 0.39 |
| Clinical characteristics |  |  |  |
| Clinical Chorioamnionitis, n (%) | 16 (16%) | 35 (17.3%) | 0.78 |
| Confirmed sepsis, n (%) | 1 (1%) | 11 (5.4%) | 0.01* |
| Days until discharge, days, median (SD) | 74 (34) | 75 (31) | 0.81 |
| Clinical measures of care |  |  |  |
| Surgery, n (5) | 0 (0%) | 5 (2.4%) | 0.02* |
| Antenatal corticosteroids, n (%) | 11 (11%) | 25 (12.3%) | 0.38 |
| Days of ETT ventilation, days, median (SD) | 7.6 (10.1) | 11.1 (12.9) | 0.04* |
| Days of CPAP, days, median (SD) | 18.1 (13.1) | 17.9 (13.5) | 0.89 |
| Hours of oxygen therapy, hours, median (SD) | 274.6 (390.2) | 282.8 (416.6) | 0.88 |
| Days of TPN, days, median (SD) | 11.0 (5.2) | 11.6 (7.1) | 0.45 |
| Hours of phototherapy, hours, median (SD) | 73.9 (53.7) | 62.7 (49.9) | 0.10 |

CA, corrected age; CPAP, Continuous Positive Airway Pressure; ETT, Endotracheal Tube; GMs, General Movements assessment; NSMDA, Neuro-Sensory Motor Developmental Assessment; PMA, Postmenstrual age; SD, Standard deviation; TEA, Term Equivalent Age; TPN, Total Parenteral Nutrition.

Supplementary Table 2. Univariate correlation as measured by the Pearson’s r correlation (and p-value) between the eight structural volumes obtained from the developing Human Connectome Project (dHCP) structural pipeline, and Bayley motor, cognitive and language composite scores. Significant correlations (p<0.05 uncorrected) are shown with an asterisk ‘*’ and are in bold.

|  | **Bayley motor composite score** | | **Bayley cognitive composite score** | | **Bayley language composite score** | |
| --- | --- | --- | --- | --- | --- | --- |
| MRI measure | Early | TEA | Early | TEA | Early | TEA |
| Extracerebral CSF | **-0.22 (0.03*)** | -0.17 (0.07) | **-0.20 (0.04*)** | -0.14 (0.15) | -0.13 (0.17) | **-0.20 (0.04*)** |
| Cortical grey matter | 0.11 (0.25) | -0.04 (0.69) | 0.18 (0.08) | -0.03 (0.79) | 0.19 (0.06) | -0.11 (0.27) |
| White matter | 0.12 (0.23) | -0.03 (0.76) | 0.16 (0.10) | <0.001 (0.99) | 0.11 (0.29) | -0.09 (0.34) |
| Ventricles | **-0.24 (0.01*)** | -0.16 (0.10) | -0.17 (0.08) | -0.15 (0.13) | -0.05 (0.60) | -0.05 (0.65) |
| Cerebellum | 0.03 (0.79) | -0.11 (0.27) | 0.01 (0.87) | -0.09 (0.36) | 0.06 (0.58) | **-0.20 (0.04*)** |
| Deep grey matter | 0.18 (0.07) | -0.08 (0.39) | 0.17 (0.10) | -0.10 (0.31) | 0.17 (0.09) | **-0.21 (0.04*)** |
| Brainstem | 0.14 (0.15) | -0.01 (0.89) | 0.11 (0.27) | <0.001 (0.99) | 0.18 (0.07) | -0.09 (0.33) |
| Hippocampus/amygdala | -0.03 (0.73) | -0.06 (0.54) | 0.04 (0.69) | 0.02 (0.88) | 0.05 (0.65) | -0.07 (0.46) |
| Global CT | -0.07 (0.49) | -0.15 (0.14) | 0.01 (0.91) | -0.15 (0.13) | <0.001 (0.99) | -0.08 (0.44) |
| Global GI | -0.04 (0.67) | -0.02 (0.84) | 0.08 (0.43) | -0.02 (0.88) | 0.08 (0.40) | 0.03 (0.76) |
| Global SA | 0.06 (0.57) | -0.06 (0.59) | 0.14 (0.17) | 0.03 (0.76) | 0.11 (0.29) | 0.04 (0.71) |
| Global SD | 0.18 (0.08) | 0.14 (0.17) | 0.09 (0.37) | 0.12 (0.23) | 0.06 (0.57) | **0.21 (0.04*)** |

CSF; CT, Cortical Thickness; GI, Gyrification Index; SA, Surface Area; SD, Sulcal Depth.

Supplementary Table 3. Correlations between the best performing models and the test set assessment scores unseen by the model, using only brain volumes as features (i.e. excluding measures of cortical shape). Bold indicates the timepoints with best statistically significant prediction accuracy.

|  | Early MRI | | Term MRI | | Combined | |
| --- | --- | --- | --- | --- | --- | --- |
|  | Pearson’s r | MAE | Pearson’s r | MAE | Pearson’s r | MAE |
| Bayley motor composite | **0.460* (p=0.0006)** | **17.96** | 0.398 (p=0.05) | 19.24 | 0.449 (p=0.009) | 28.61 |
| Bayley cognitive composite | **0.518* (p=0.001)** | **19.19** | 0.475* (p=0.003) | 20.47 | **0.519* (p=0.007)** | **23.42** |
| Bayley language composite | 0.302 (p=0.001) | 24.72 | 0.416 (p=0.008) | 28.32 | 0.311 (p=0.009) | 24.88 |
| NSMDA functional grade | 0.463* (p=0.003) | 3.28 | 0.477* (p=0.002) | 3.20 | **0.506** (p<0.0001)** | **2.44** |

**p*<0.004, ***p*<0.0001. MAE, Mean Absolute Error; MRI, Magnetic Resonance Images; NSMDA: Neuro-sensory Motor Developmental Assessment.

Supplementary Figure 1. Scatter plots illustrating the univariate correlation between structural volumes (early ‘30w’ above, term equivalent age ‘40w’ below) obtained using the developing Human Connectome Project (dHCP) structural pipeline, and Bayley motor, cognitive and language composite scores.
